# Sex-specific differences of amlexanox in a mouse model for atherosclerosis and MASLD

**DOI:** 10.1016/j.molmet.2026.102426

**Published:** 2026-07-21

**Authors:** Eleonora Mungo, Michelle Haß, Denis Benning, Tobias Schmid, Silvia Kuntschar, Sofie P. Meyer, Lisa Hahnefeld, Erika Dorochow, Urs Christen, Edith Hintermann, Rebekka Medert, Marc Freichel, Gerd Geisslinger, Ellen Niederberger

**Affiliations:** 1 Goethe-University Frankfurt, Faculty of Medicine, pharmazentrum frankfurt/ZAFES, Institute of Clinical Pharmacology, Theodor Stern Kai 7, Frankfurt am Main, 60590, Germany; 2 Goethe-University Frankfurt, Faculty of Medicine, Institute of Biochemistry I, Theodor Stern Kai 7, Frankfurt am Main, 60590, Germany; 3 Goethe-University Frankfurt, Faculty of Medicine, pharmazentrum frankfurt/ZAFES, Institute of General Pharmacology and Toxicology, Theodor Stern Kai 7, Frankfurt am Main, 60590, Germany; 4 Institute of Pharmacology, Ruprechts-Karl University Heidelberg, Im Neuenheimer Feld 366, Heidelberg, 69120, Germany; 5 Fraunhofer Institute for Translational Medicine and Pharmacology ITMP, Sandhöfer Allee 1, Frankfurt am Main, 60528, Germany

**Keywords:** Atherosclerosis, Fatty liver disease, PCSK9, IKKε, Amlexanox

## Abstract

**Objectives:**

Inhibitor-κB kinase epsilon (IKKε) is a non-canonical IκB kinase involved in NF-κB signaling and type I interferon responses. We recently demonstrated sex-dependent effects of IKKε deletion on atherosclerosis and metabolic dysfunction-associated steatotic liver disease (MASLD), with male knockout mice showing protection against both diseases, while female mice exhibited exacerbated inflammatory and metabolic disturbances. These divergent outcomes were linked to differential effects on inflammatory pathways and lipid metabolism.

**Methods:**

To evaluate the therapeutic potential of pharmacological IKKε inhibition, we treated wild type mice with established atherosclerotic plaques and hepatic steatosis - induced by PCSK9 gain-of-function and Paigen diet - with the IKKε inhibitor amlexanox.

**Results:**

Amlexanox modulated serum lipid levels and altered plaque composition but did not halt plaque progression. In the liver, treatment produced marked sex-specific effects: male mice exhibited substantial improvement in steatosis, whereas female mice showed worsened lipid accumulation. These outcomes were reflected in pronounced sex-dependent differences in serum and hepatic lipid and metabolite profiles, indicating regulation of fatty acid and bile-acid metabolism predominantly in males. Protein analyses in liver and adipose tissue further supported opposing metabolic and inflammatory responses between sexes after amlexanox treatment.

**Conclusions:**

Collectively, our findings indicate that therapeutic IKKε inhibition with amlexanox does not prevent progression of advanced atherosclerosis in this model but effectively ameliorates MASLD in male mice. In contrast, female mice experience aggravated hepatic lipid deposition. These results underscore the importance of incorporating sex-specific analyses in metabolic and cardiovascular research and highlight the need to evaluate therapeutic strategies such as amlexanox in both sexes.

## Highlights

• Amlexanox has no impact on already established atherosclerotic plaques.
• In male mice, amlexanox attenuates Paigen-diet-induced MASLD.
• Amlexanox modulates serum and liver lipids and metabolites.
• Amlexanox reduces fatty acid synthesis and de novo lipogenesis in male mice.
• Female mice are not affected by amlexanox treatment.

## 1 Introduction

A Western lifestyle, particularly excessive nutrient intake, is associated with chronic diseases such as cardiovascular and metabolic disturbances, including atherosclerosis and metabolic dysfunction-associated steatotic liver disease (MASLD) [[1], [2], [3]]. Both conditions frequently emerge on the basis of metabolic syndrome features—insulin resistance, dyslipidemia, and hypertension—and often occur concurrently. In patients with MASLD, cardiovascular disease (CVD) represents the leading cause of mortality [[4], [5], [6]] and increasing evidence indicates that MASLD is a risk factor for development of atherosclerosis [[5], [6], [7]]. Mixed lipidemia and hypercoagulability, particularly elevated low-density lipoproteins (LDL) and enhanced oxidative processes, are proposed as shared drivers of both diseases [[8], [9], [10]]. Although several therapeutic options exist for CVD, pharmacological treatments for MASLD and metabolic dysfunction-associated steatohepatitis (MASH) remain limited. Current management relies primarily on lifestyle modification, including dietary changes and increased physical activity, which is difficult to sustain long term. Pharmacological agents targeting comorbidities, such as GLP-1 receptor agonists, SGLT2 inhibitors, statins, and vitamin E, are recommended but often show modest efficacy. The thyroid hormone receptor agonist resmetirom was approved by the FDA in 2024 as the first drug to improve metabolic syndrome and non-alcoholic steatohepatitis (MASH). However, long-term safety and efficacy data are still lacking [11,12]. Consequently, there is a necessity to develop further effective pharmacological interventions.

In addition to dysregulation of lipid metabolism, low-grade non-resolving inflammation is a major contributor to obesity, MASLD and atherosclerosis [13,14]. The non-canonical I-κB kinase epsilon (IKKε) is rapidly upregulated in response to inflammatory stimulation [15,16] and has been previously linked to obesity and CVD [17,18]; however, the findings are not entirely consistent. In most studies, inhibition of ΙΚΚε, either through genetic deletion or pharmacological treatment with amlexanox, improves insulin sensitivity, glucose tolerance, and hepatic steatosis [17,[19], [20], [21]]. A number of studies have indicated that these effects are attributable to increased energy expenditure, decreased inflammation and enhanced catecholamine sensitivity [[20], [21], [22]]. Our recent work demonstrated that IKKε deletion reduces serum lipid levels and inhibits the initiation and progression of both atherosclerosis and MASLD in the PCSK9 gain-of-function (GOF)/high-fat diet (HFD) model, with more pronounced effects in male mice due to sex-specific molecular regulation in liver and adipose tissue [19]. Two actual studies reported that amlexanox reduces plaque size and improves hepatic function in male LDL-R^−/−^mice [18,23]. In the present study, we evaluated the therapeutic efficacy of amlexanox in the PCSK9-GOF/HFD model in wild-type mice of both sexes. In contrast to complete IKKε deletion, late-onset amlexanox treatment did not reverse aortic plaques. However, in MASLD, we observed a sex-dependent improvement in liver morphology, which was restricted to male mice.

## 2 Materials and methods

### 2.1 Drugs

Amlexanox used as IKKε inhibitor [21] was purchased from Cayman Chemical (Biomol, Hamburg, Germany, CAS68302-57-8). The drug was dissolved in 100% DMSO at a concentration of 10 mg/ml. For animal experiments, a dose of 25 mg/kg body weight was used (according to [21]). The corresponding volume of DMSO was administered as vehicle. Amlexanox concentrations in the serum of mice were determined as described previously [24].

### 2.2 Mice

Wild type C57BL/6J were ordered from Charles River Laboratories (Sulzfeld, Germany). In all experiments, almost equal numbers of male and female mice were used. The treatment started at the age of 6–8 weeks. Animals were maintained in climate- and light-controlled rooms (24 ± 0.5 °C, 12/12 h dark/light cycle) and had free access to food and water during the whole observation time. In all experiments, the European ethic guidelines for investigations in conscious animals were obeyed and the procedures were approved by the local Ethics Committee for Animal Research (Regierungspräsidium Darmstadt FK/1004 and FK/1115). All efforts were made to minimize animal suffering and to reduce the number of animals used (in compliance with the ARRIVE and the Directive 2010/63/EU guidelines).

### 2.3 PCSK9 GOF and high-fat diet model

Adeno-associated viral vectors encoding the gain-of-function variant D377Y of the murine PCSK9 (rAAV8-PCSK9D377Y) under the control of a liver-specific promoter were kindly provided by the Institute of Pharmacology, University of Heidelberg. The viral particles (1.0 × 10^11^ viral genomes per mouse) were administered via injection into the tail vein of mice. Control animals received an i.v. injection with 0.9% NaCl. Afterwards, the mice were fed with a high-cholesterol/high-fat Paigen diet (PD, containing 16% fat, 1.25% cholesterol, and 0.5% sodium cholate (Ssniff, Germany)) to induce chronic hypercholesterolemia. After 8 weeks of diet feeding, blood was collected by puncture of the retrobulbar venous plexus to analyse lipid concentrations before the treatment with amlexanox (25 mg/kg BW, p.o.) or vehicle (DMSO) was started. Animals were treated daily for 5 days/week and the PD was proceeded for additional 8 weeks.

During the first 8 weeks of treatment, the body weight of the animals was determined at least once per week. After the start of drug treatment, mice were weighed on every treatment day until the end of the experiment. Some mice (∼4%) showed an inability to tolerate the Paigen Diet during the initial four weeks period of the study. They exhibited a strong reduction in body weight, which was identified as a termination criterion. These mice were excluded from further analysis. At the end of the treatment period, mice were sacrificed, blood was collected and aorta, liver and lipid tissue were excised.

### 2.4 Determination of blood parameters

At the end of the experiment, the animals were sacrificed and blood was withdrawn by cardiac puncture. Serum samples were generated by clotting for 10 min and subsequent centrifugation at 2,000×*g* for 20 min at room temperature without braking. The serum from the upper phase was rapidly frozen in liquid nitrogen and then stored at −80 °C until further analysis.

For determination of blood parameters (total cholesterol, LDL, HDL, triglycerides, aspartate transaminase (AST), glucose), the serum samples were diluted 1:3 with 0.9% NaCl and the analyses were performed in the central laboratory of the University Clinic Frankfurt.

### 2.5 Determination of total bile-acid

Total Bile-acid in liver and serum was analyzed using a Colorimetric Mouse Total Bile-acids Assay Kit (CrystalChem, Itasca, IL, USA #80471) according to the manufacturer's recommendations. Liver homogenates and serum were diluted 1:10 prior to analysis.

### 2.6 Analysis of polar metabolites and lipids using LC-HRMS

A detailed description of the methods can be found in previous publications [19,25,26]. Briefly, for homogenization of liver tissue, tissue pieces were homogenized in varying volumes of pre-cooled extraction solution (25% ethanol + 10 μM indometacin), depending on the individual tissue weights. Homogenization was performed by wet grinding using a Precellys 24-Dual tissue homogenizer coupled with a Cryolys cooling module (both Bertin Technologies, Montigny-le-Bretonneux, France). Lipids and polar metabolites were extracted from liver homogenates (0.05 mg/μL) via liquid–liquid extraction using 20 μL of tissue homogenate, corresponding to 1 mg of tissue material. Samples were kept in ice water during processing and the extracted and dried samples were stored at <−70 °C until analysis. Just before analysis, the dried organic layer was reconstituted with 100 μL of methanol while the dried water layer was resuspended in 100 μL of 50% acetonitrile for the profiling of lipids and polar metabolites, respectively. QC samples were created from equivalent volumes of homogenized samples that were processed the same day. For serum analysis, 10 μL of serum were combined with 75 μL of internal standard solution in methanol (MeOH), 250 μL of methyl *tert*-butyl ether (MTBE), and 50 μL of 50 mM ammonium formate. The mixture was vortexed and centrifuged at 20,000×*g* for 5 min at 4 °C. The upper phase was transferred to a vial for lipidomics analysis. The lower phase was re-extracted with 100 μL of a saturated MTBE solution (MTBE/MeOH/H_2_O, 10:3:2.5), vortexed, and centrifuged again for 5 min at 4 °C. The upper phase from this step was combined with the initial upper phase in the vial. The combined upper phases were evaporated under nitrogen at 45 °C and reconstituted in 100 μL of MeOH for lipidomic analysis. For the polar metabolite analysis, the lower phase was transferred to a glass vial, and any remaining upper phase residue was discarded, before evaporation under nitrogen at 45 °C. The dried residue was reconstituted in 100 μL of 50% acetonitrile. The study utilized a Vanquish Horizon UHPLC system paired with an Orbitrap Exploris 480 mass spectrometer (Thermo Fisher Scientific, Dreieich, Germany). Lipids were separated using a Zorbax RRHD Eclipse Plus C8 column (50 mm × 2.1 mm ID, 1.8 μm particle size) and a same-type pre-column (Agilent Technologies, Waldbronn, Germany). A 14-minute binary gradient with water (0.1% formic acid (FA), 10 mM ammonium formate) as eluent A and acetonitrile:isopropanol (2:3, v/v) with 0.1% FA as eluent B was employed. For polar metabolites, a SeQuant ZIC-HILIC column (100 mm × 2.1 mm ID, 3.5 μm particle size) with a same-type pre-column (Merck, Darmstadt, Germany) was utilized, and separation was achieved using a binary gradient of water (0.1% FA) as eluent A and acetonitrile (0.1% FA) as eluent B. System operation was managed via XCalibur software v4.4 and Tune Application 3.1 (Thermo Fisher Scientific, San Jose, USA). Data analysis was conducted using TraceFinder software v5.1 (Thermo Fisher Scientific, San Jose, USA). Identification of compounds was performed using the mzCloud offline library 2020 and LipidBlast VS68 libraries. A heated electrospray ionization (H-ESI) source facilitated MS data acquisition in full scan mode at a resolution of 120,000. MS2 spectra were obtained in a data-dependent manner (ddMS2) with a resolution of 15,000 and a cycle time of 600 ms.

Normalization of lipid results was done using one internal standard per lipid class, while polar metabolite results were normalized with probabilistic quotient normalization. Due to insufficient sample material for a QC pool, system performance was verified using a mixture of human plasma QCs and reinjections of a single sample. Reporting molar concentrations based on the IS concentrations was omitted in the lipid analysis, given that the method employed (reversed-phase chromatography) enables robust relative quantification. However, it is important to note that the reported values should not be regarded as absolutely quantitative (μmol/mL). To provide a visual representation of this data, the peak area ratios are presented.

### 2.7 Histology

The livers as well as the aortic arches including brachiocephalic arteries were prepared, subjected to 2% PFA for 2 h and dehydrated in 20% sucrose-solution for 2h and then in 30%-sucrose solution overnight at 4 °C. Afterwards, they were embedded in O.C.T. compound (Sakura Finetek Europe B.V., Alphen aan den Rijn, Netherlands). These cryo-embedded tissues were cut into 8 μm slices in a cryotome (Leica CM3050S). Several aortas were also used for *en-face* analysis for determination of the number of plaques.

Frozen sections of aorta and liver were subjected to Oil-Red O/hematoxylin (ORO/H) and Picrosirius Red staining, respectively. For lipid staining with ORO/H, sections were fixed 5 min in 4% PFA, washed 5 min in isopropanol (60%) and then stained for 10 min with ORO solution (0.6 g ORO in 120 ml isopropanol). After 4 washings in isopropanol and water, sections were stained with hematoxylin for 6 min and then again washed with tap water. Afterwards, they were mounted with Aqua-Poly/Mount (Polysciences Europe, Hirschberg, Germany). For the collagen staining, slides were incubated with Picrosirius Red solution (Sigma–Aldrich, Germany) for 1 h at room temperature and then washed twice in acidified water (5 ml glacial acetic acid to 1 L of water) and mounted.

For histological analysis, images were captured on a Keyence BZ-X810 fluorescence microscope and ImageJ software 1.54f was used for analysis. The plaque size was calculated as a ratio of the intima area/media area. ORO/H and picrosirius staining was quantified by the amount of red color in the tissues.

For *En face* staining of whole aortas, the vessels were fixed 24 h in 4% PFA at 4 °C. Then they were opened longitudinally and incubated with PBS-Triton X-100 (0.1%) at room temperature. After a washing step with PBS and 3 min incubation with isopropanol (60%), they were subjected to the ORO-solution for 13 min and rinsed again with isopropanol (60%). The stained aortas were analysed with the stereo-microscope Nikon SMZ1000 (Nikon, Japan) and NIS-Elements D software (Nikon, Japan).

### 2.8 Immunofluorescence staining

Immunofluorescence analysis of aorta cryoslices started with two washing steps with ice cold PBS for 5 min and permeabilization with PBS-Triton X-100 (0.1%) for 10 min. The sections were then blocked in 3% BSA/10% NGS in PBS for 1 h to reduce non-specific binding. After an incubation overnight at 4 °C with primary antibodies against α-SMA (smooth muscle cell marker) (Invitrogen, Thermo Scientific, Germany (PA5-85070), 1:200), and CD 86 (proinflammatory macrophage marker) (Proteintech, Germany, (13395-1-AP) 1:200), dissolved in PBS/3% BSA, slices were rinsed in PBSTx (0.1% Triton) and then incubated for 2 h at room temperature with Cy3-conjugated secondary antibodies (Sigma, Merck; Germany, (C2306), 1:1,200) dissolved in PBSTx (0.1% Triton). An additional staining with secondary antibody only served as background control. This was followed by additional staining with DAPI (5 μM in DPBS, Carl Roth GmbH + Co. KG, Germany) for 5 min. After final rinsing in PBS, the sections were mounted with Aqua-Poly/Mount (Polysciences Europe, Hirschberg, Germany). Images were captured using an inverted fluorescence microscope (Axio Observer.Z1, Zeiss, Germany) equipped with a monochrome CCD camera and AxioVision software (Zeiss, Germany). Image analysis was performed with ImageJ 1.54f software. Therefore, images were converted to 8bit B/W. Then, a threshold was set and the fluorescence signals in the plaque area quantified.

### 2.9 Immunohistological staining

For immunohistological staining, fresh cryosections of liver tissue were prepared and then fixed with cold ethanol (99.8%) (−20 °C). The slides were then washed twice for 2 min with PBS and treated for 10 min at RT with hydrogen peroxide (0.3%)/sodium azide (0.1%) solution in PBS to block endogenous peroxidases. After 2 washing steps for 2 min with PBS, the Avidin/Biotin Blocking Kit from Vector Laboratories, Inc (USA) was used according to the manufacturer's instructions. After the final wash step with PBS, non-specific protein binding sites were blocked with a 10% FBS-PBS solution for 30 min at RT followed by incubation with the primary antibodies (CD8b (BD Biosciences, 1:500, 2 h), CD11b (eBioscience ThermoFischer Scientific, 1:500, 16h), Ly6G (BioXcell, 1:500, 16 h) at 4 °C. After the end of the incubation periods, slides were washed three times with PBS for 4 min, and the biotinylated secondary antibodies were applied for 1 h at RT. After 3 washing steps with PBS, the Vectastain® Elite® ABC Reagent (Vector Laboratories, Inc., USA) was dropped onto the sections and incubated for 30 min at RT. Following 3 washing steps with PBS, staining was performed for exactly 5 min with DAB Substrate Kit, Peroxidase (HRP) from Vector Laboratories, Inc (USA). The reaction was stopped by immersing the slides in PBS. Finally, the sections were stained with hematoxilin (Carl Roth GmbH + Co. KG, Germany) for 5 min, washed twice with PBS and coverslipped with Aqua-Poly/Mount (Polyscience Inc., USA). The slides were examined microscopically using the Keyence BZ-9000 microscope (Keyence Deutschland GmbH, Germany) equipped with BZ-II Viewer and BZ-II Analyzer software.

### 2.10 Western Blot analysis

Liver and adipose tissue samples were homogenized in PhosphoSafe Extraction Buffer (Merck, Darmstadt, Germany) containing protease inhibitor (1 mM Pefabloc SC, Alexis Biochemicals, Lausen, Switzerland) using an Ultrathurrax instrument (T10 basic, VWR, Germany). Samples were kept at room temperature for 3 min before the cell lysate was centrifuged at 14,000 rpm for 30 min at 4 °C in an Eppendorf centrifuge. The protein-containing supernatant was stored at −80 °C until further analysis.

Proteins (30 μg) were separated electrophoretically by 10, 12 or 16% SDS-PAGE and then transferred onto nitrocellulose membranes by semidry-blotting (Bio-Rad, München). To control the quality of the transfer, all blots were stained with Ponceau red solution. Membranes were blocked for 60 min at room temperature in Odyssey blocking reagent (LI-COR Biosciences, Bad Homburg, Germany) diluted 1:2 in 0.1 M PBS, pH 7.4. Afterwards, the blots were incubated overnight at 4 °C with primary antibody against pACC (#3661), ACC (#3676), ΙΚΚε (#3416), TBK1 (#3013), SCD1 (#2794), FABP1(#13368), FASN (#3180), FXR/NR1H4 (#72105),(all 1:250, Cell Signaling Technology, Boston, USA), LPL (1:500, Thermo Scientific, Darmstadt, Germany (MA535444)), SREBP (1:500, Thermo Scientific, Darmstadt, Germany, (#PA1-337)), CRP (1:500, Cloud-Clone Corp., Kati, TX, USA (# PAA821Mu01)), in Odyssey blocking reagent diluted 1:2 in 0.1% Tween 20 in 0.1 M PBS. After washing three times with 0.1% Tween 20 in 0.1 M PBS, the Blots were incubated for 60 min with an IRDye 680-conjugated secondary antibody (LI-COR, Bad Homburg, Germany, (926-68071)), 1:10,000 in blocking buffer diluted 1:2 in 0.1% Tween 20 in 0.1 M PBS). After rinsing in 0.1% Tween 20 in 0.1 M PBS, protein-antibody complexes were detected with the Odyssey Infrared Imaging System (LI-COR Biosciences). β-actin (37 kDa) (1:1,200, Sigma, Germany (#A5441)) was used as loading control and detected with an IRDye 800-conjugated secondary antibody (LI-COR, Bad Homburg, Germany, (926-32210)). Densitometric analysis of the blots was performed with Image Studio Lite Software (LI-COR, Biosciences).

### 2.11 Cytometric bead array (CBA)

IL10 and TNFα concentrations in liver samples were quantified using cytometric bead array LEGENDplex (BioLegend) according to the manufacturer's instructions. Samples were measured by flow cytometry and subsequently analysed using FlowJo software V10 (Treestar, Ashland, OR, USA).

### 2.12 Real-time PCR

RNA was prepared from mouse livers and fat tissue using TRI reagent as described previously [26]. Two hundred nanogram of total RNA was used for the reverse transcription, which was performed with Random and Oligo-dT Primers (2:1 ratio) in a Verso cDNA Synthesis Kit (Thermo Scientific, Darmstadt, Germany). Twenty nanogram RNA equivalents were subjected to real-time PCR in a QuantStudio 5 Real-Time PCR system using the SYBR Select Master Mix (Rox) (Life Technologies, Austin, USA). Expression of mRNA was assessed related to GAPDH mRNA.

The following gene-specific primers were used:

CD206: FW 5′-CATCGAGACTGCTGCTGAGT-3′RV 5′-ACCAAAGCCACTTCCCTTCA-3′

Cyp7a: FW 5′- GGGATTGCTGTGGTAGTGAGC-3′RV 5′- GGTATGGAATCAACCCGTTGTC-3′

FXR/NR1H4: FW 5′-AGGGGATGAGCTGTGTGTTG-3′ RV 5′-ACACTTGTACACGGCGTTCT-3′

IL-1β: FW 5′- GCAACTGTTCCTGAACTCAAC-3′RV 5′- ATCTTTTGGGGTCCGTCAACT-3′

IL-10: FW 5′-GCTCTTACTGACTGGCATGAG-3′RV 5′-CGCAGCTCTAGGAGCATGTG-3′

PPARα: FW 5′- AACATCGAGTGTCGAATATGTGG-3′RV 5′- CCGAATAGTTCGCCGAAAGAA-3′

GAPDH: FW 5′-CAA TGT GTC CGT CGT GGA TCT-3′RV 5′-GTC CTC AGT GTA GCC CAA GAT G-3′

The cycle number at which the fluorescence signal crosses a defined threshold (Ct-value) is proportional to the number of RNA copies present at the start of the PCR. The threshold cycle number for the specific mRNA was standardized by subtracting the Ct-value of GAPDH from the Ct-value of gene-specific amplificates of the same sample, respectively. Relative quantitative level of samples was determined by standard 2^-(ddCt)^ calculations and expressed as fold-change of a single reference control sample.

### 2.13 RNA-seq of liver samples, functional annotation and pathway analysis

RNA was extracted from liver tissue using TRI Reagent as indicated above. The RNA concentration as well as the quality and integrity of RNA were controlled using RNA ScreenTape assays on a TapeStation 4150 (Agilent Technologies, Waldbronn, Germany) and Qubit RNA HS Assay Kits on a Qubit 3.0 Fluorometer (Thermo Fisher Scientific). Sequencing libraries were prepared according to the workflow of the Quant Seq 3’ mRNA-Seq V2 Library Prep Kit FWD with UDI (Lexogen, Vienna, Austria). During the process, an additional step was taken to calculate the optimal cycle number for the end-point PCR. For this, the PCR Add-on Kit V2 for Illumina (M02096-2-0130) was used. The quality of cDNA libraries was assessed using HS-D1000 ScreenTape assays on a TapeStation 4150, and quantities were measured using Qubit dsDNA HS Assay Kits. Libraries were sequenced (single end, 75 cycles) using a P2 100-cycle kit on a NextSeq 2000 instrument (Illumina, San Diego CA, USA).

To assign the samples to their ID, the client BaseSpace Sequence Hub from Illumina was used for demultiplexing. The data was analyzed using Lexogens own data analysis pipeline now called Kangaroo, which was previously performed via the BlueBee genomics platform at that time the RNA-sequencing was performed. Gene set enrichment analysis (GSEA) was performed using the GSEA module (version 4.4.0) [27] on the GenePattern platform [28].

### 2.14 Statistical analysis

Statistical analyses were performed with Graph Pad Prism (version 10; Graph Pad Software Inc., La Jolla, CA, USA). The significance level was set at P < 0.05 for all comparisons. Data are presented as mean ± standard deviation (SD). For all data, the D'Agostino-Pearson normality test was used. Comparison of two groups were analyzed either with unpaired Student's t-test or Mann Whitney nonparametric test. Statistical analyses of more than two groups were performed with analysis of variance (ANOVA), and Tukey's multiple comparisons test.

## 3 Results

### 3.1 The impact of amlexanox on weight gain, serum lipid levels and atherosclerotic plaques

**Figure 1:**
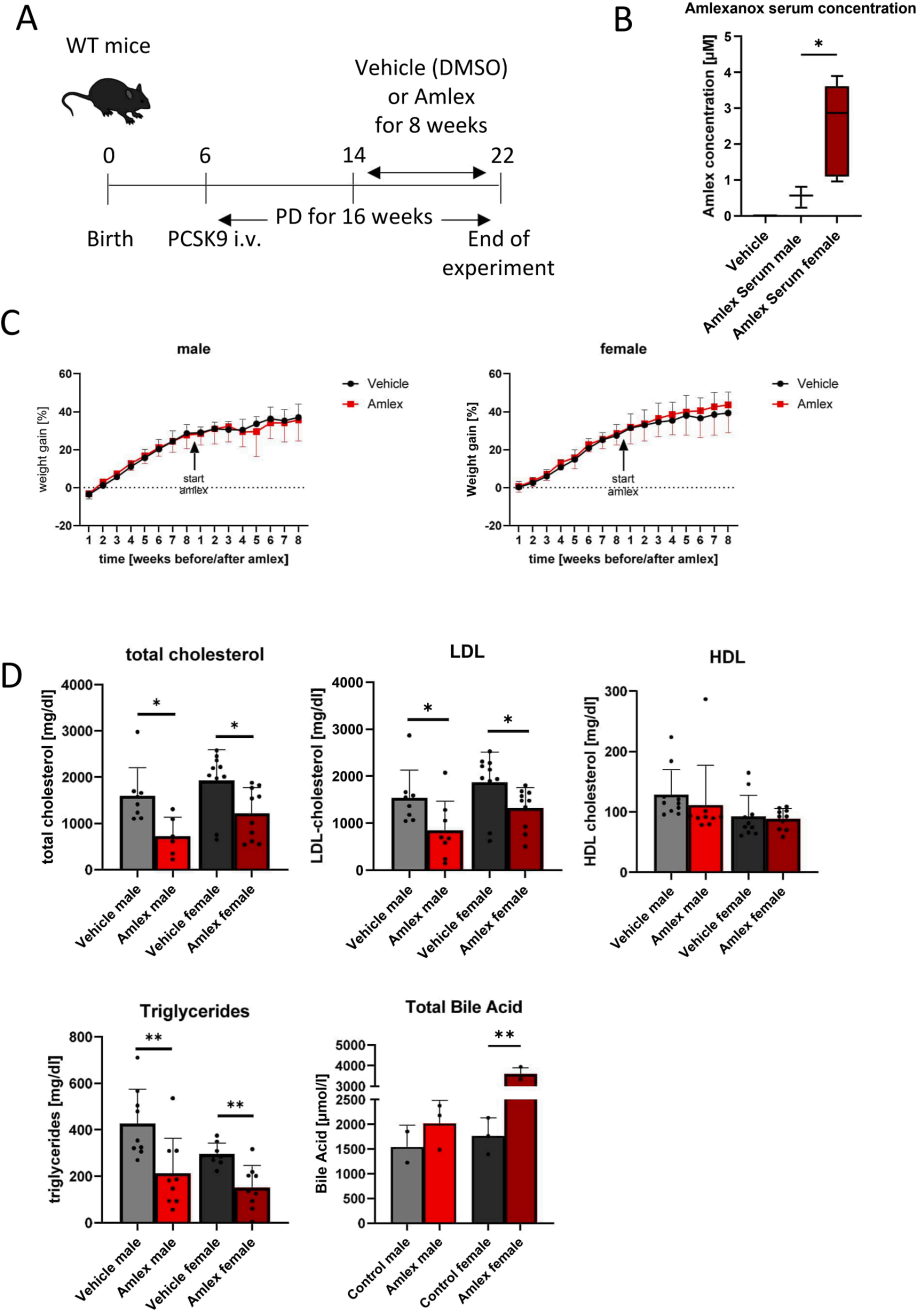
Weight gain, amlexanox serum concentration and serum lipids in wild type mice treated with vehicle or amlexanox (A) Schematic overview of the treatment regimen of wild type mice with vehicle (DMSO) and amlexanox, (B) amlexanox serum concentrations 2 h after the last oral administration of the drug (n = 3–5 mice/group), (C) weight gain of male and female mice eight weeks before and eight weeks after the start of vehicle or amlexanox treatment (n = 7–9 mice/group), (D) serum lipid levels of male and female vehicle- or amlexanox-treated mice (n = 8–10 mice/group), Student's t-test for comparison of DMSO and amlexanox in male and female mice, respectively, ∗*P* < 0.05, ∗∗*P* < 0.01.

**Figure 2:**
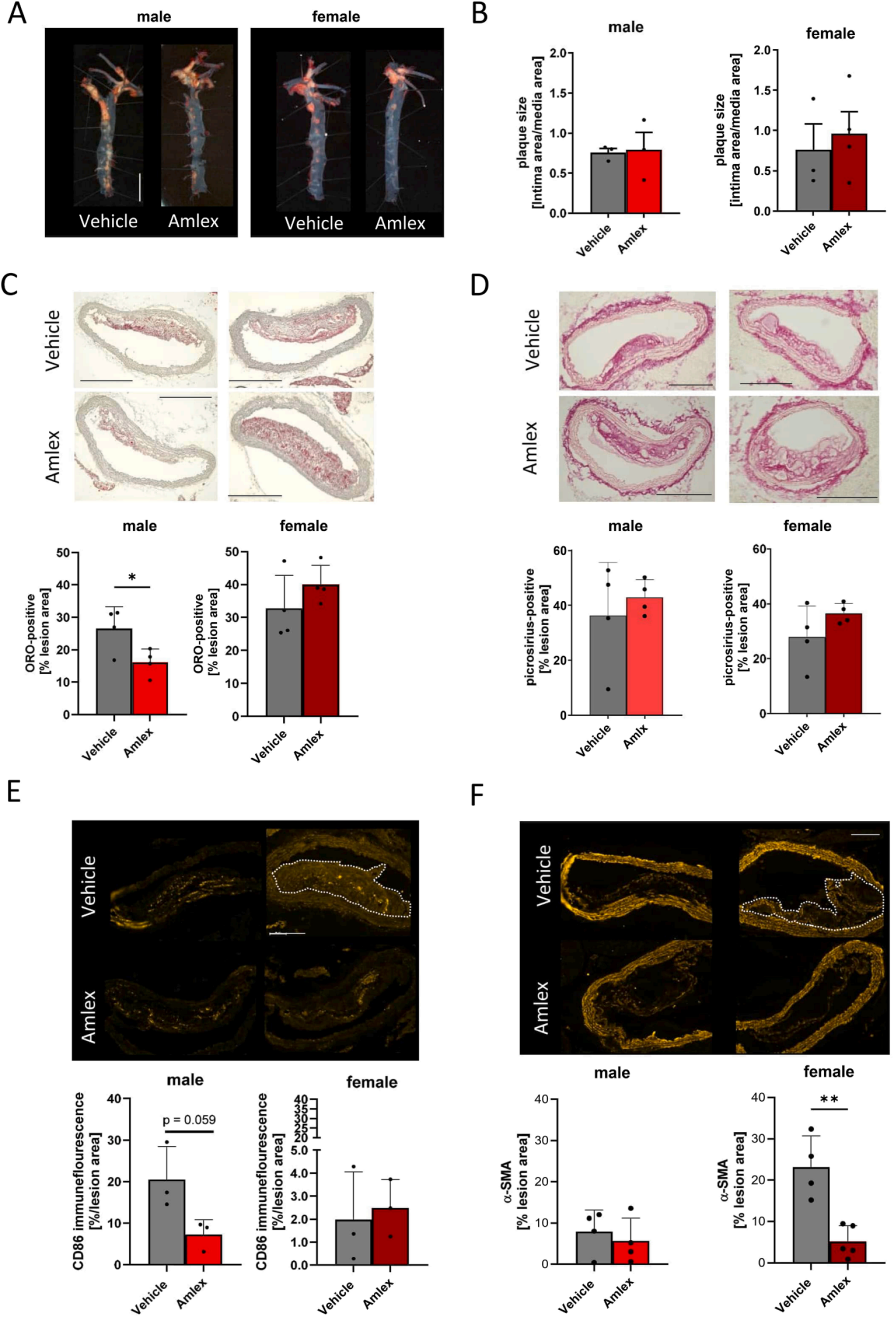
Plaque size and characterization in wild type mice treated with vehicle or amlexanox (A) *En face* staining with ORO in aortas of male and female mice treated with vehicle or amlexanox; (scale bar: 5 mm), (B) quantitative analysis of the plaque size in H&E-stained cross sections (Ratio intima area/media area), (C) ORO/H-staining of aorta cross sections and quantitative analysis, (D) Picrosirius Red stain and quantitative analysis (B and D scale bar: 250 μm), (E, F) immunofluorescence staining and quantitative analysis for (E) CD86 and (F) αSMA in the plaques (E and F scale bar: 200 μm), representative pictures of at least three independent experiments, the analysed plaque area is representatively indicated by the dotted white line (n = 3–5/group), Student's t-test. ∗p < 0.05, ∗∗p < 0.01.

**Fig. S1:**
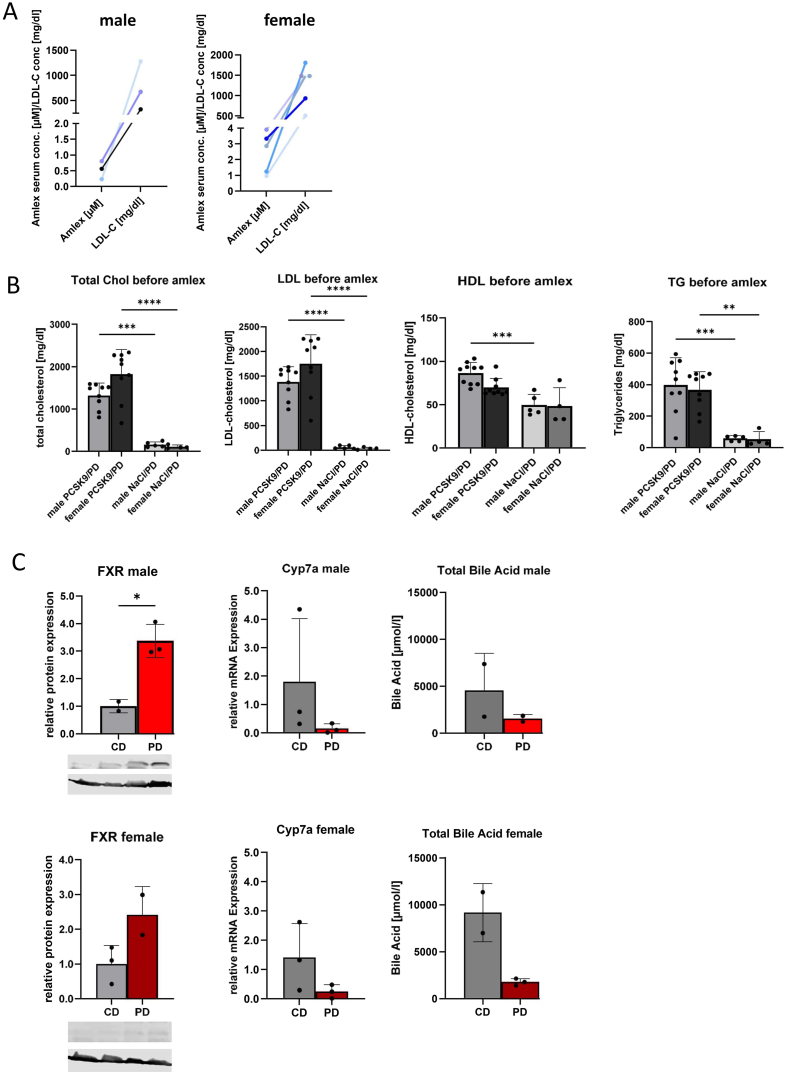
***Suppl. Figure 1:****Analysis of serum and liver parameters*(A) Correlation of serum amlexanox concentration with serum LDL-cholesterol levels in male and female mice (n=3 for male, n=4 for female mice). (B) Total Cholesterol, LDL- and HDL-cholesterol and triglycerides in the serum of female and male wildtype mice after PCSK9 injection and eight weeks of Paigen diet. Control animals received a NaCl injection and Paigen diet for eight weeks. The injection of PCSK9 induced significantly higher lipid levels compared to NaCl-treated mice. (n= 4-9/group), One-way ANOVA, ∗∗ p < 0.01,∗∗∗ p < 0.001,∗∗∗∗ p < 0.0001. (C) FXR protein level assessed by Western Blot analysis, Cyp7a mRNA expression in the liver and serum total bile-acid levels of male and female control mice treated with either control (CD) or Paigen diet (PD). Student’s t-test, ∗p < 0.05 (n = 2-3/group)

Wild type mice of both sexes were either injected with AAV8-PCSK9-GOF (adeno-associated virus-8 (AAV8)-PCSK9^D377Y^) or 0.9% NaCl and subsequently fed with a high fat diet (Paigen Diet, PD) over a period of 16 weeks. At eight weeks of the diet feeding, treatment with amlexanox or a vehicle control (DMSO) was initiated and continued for a further eight weeks (Figure 1A) to gain insight if amlexanox is able to reverse the effects of PCSK9/PD-induced dysregulations. At the end of the experiment, serum amlexanox concentrations were measured, yielding levels of 0.461 ± 0.236 μM in treated male mice and significantly higher concentrations of 2.454 ± 1.058 μM in females indicating sex-specific differences in pharmacokinetics. These values fall within the magnitude of the reported IC_50_ of approximately 1 μM for IKKε inhibition in vitro. Notably, the serum concentrations did not directly correlate with the amlexanox-induced effects (Suppl. Figure 1A). In mice treated with vehicle, amlexanox was not detectable (Figure 1B). During the 16-week treatment period, both male and female mice demonstrated comparable weight gain, with no discernible differences observed between the DMSO and amlexanox treatment groups (Figure 1C). Serum lipid concentrations were analysed initially after eight weeks of PD only, and subsequently at the end of the experiment, to ascertain the individual differences of each mouse after DMSO or amlexanox feeding, respectively. All mice treated with PCSK9/PD exhibited elevated levels of total cholesterol (TC), LDL-C, and triglycerides after the first eight weeks of treatment in comparison to control mice that received NaCl/PD (Suppl. Figure 1B). Because the PCSK9/PD model elevates cholesterol not only through PCSK9-GOF–mediated LDL-R reduction but also through diet-induced FXR activation, which suppresses Cyp7a and bile-acid synthesis [29,30], we additionally analyzed these pathways. Paigen diet increased hepatic FXR expression, while Cyp7a and serum bile-acid concentrations were reduced (Suppl. Figure 1C). The oral administration of amlexanox led to a significant reduction of the PCSK9/PD-induced increase in TC, LDL-C and triglyceride levels in both male and female mice. In contrast, bile-acid levels raised and HDL was found to be unaltered in both sexes (Figure 1D). The development of aortic plaques was assessed through two methods: *en-face* staining with Oil Red O (ORO) and cross-sectional staining with H&E. *En-face* staining showed no significant differences in the number of atherosclerotic plaques in mice treated with amlexanox or vehicle. In addition, quantitative analysis of the plaque size in cross-sections revealed no differences between vehicle-treated and amlexanox-treated groups in both male and female mice (Figure 2 A and B).

Further histological analysis with ORO/H staining showed a decrease in fat deposition in the plaques of male mice treated with amlexanox (Figure 2C) while collagen staining with Sirius red revealed no differences between the groups (Figure 2 D). In immunofluorescent stainings, male control mice showed a higher number of CD86-positive cells, e.g. macrophages in the plaques than female mice, which however, were strongly reduced after treatment with amlexanox (Figure 2 E). Furthermore, a decline of alpha smooth muscle actin (αSMA) in the plaques of female mice was observed (Figure 2 F).

### 3.2 Effects of amlexanox on liver and adipose tissue

**Figure 3:**
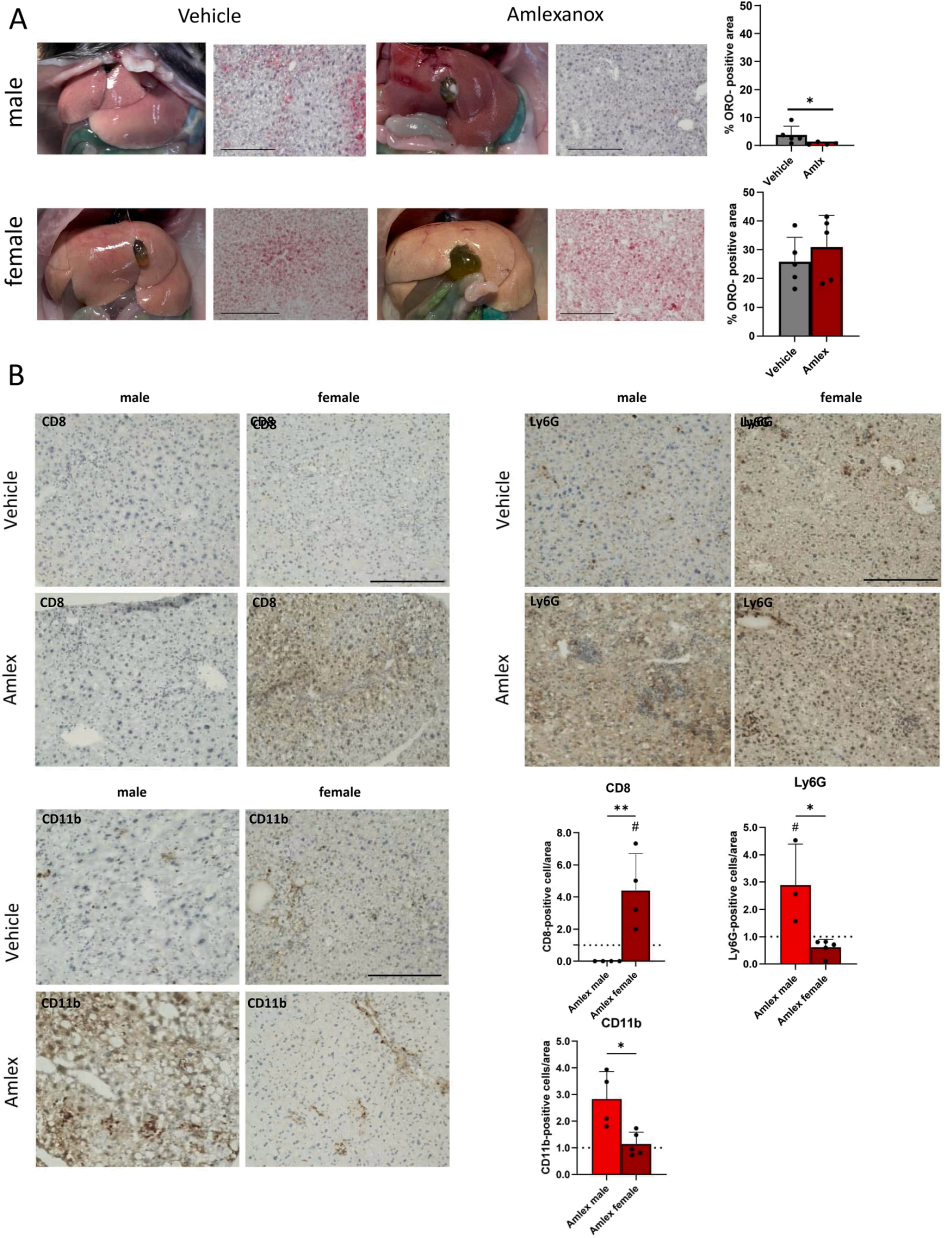

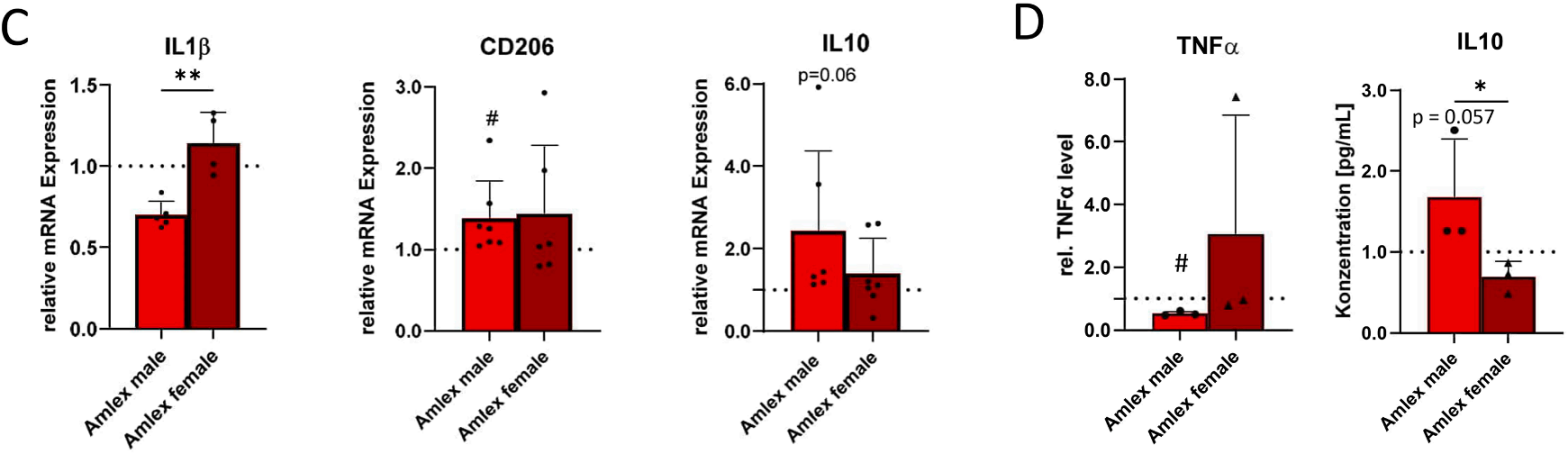
Effects of amlexanox treatment on MASLD Effects of vehicle or amlexanox in livers of PCSK9/PD-treated male and female wild type mice. (A) Liver morphology and histology of wild type mice treated with PCSK9/PD and vehicle or amlexanox. The histological stainings were performed with haematoxylin and Oil red O in liver slices of the respective mice. Quantitative analysis of n = 4–5/group. Student's t-test. ∗p < 0.05, (B) Immunohistological staining for CD8 T-cells, CD11b-positive leukocytes and Ly6G-positive neutrophils with quantitative analysis (n = 4–5/group), 2-way ANOVA, ∗p < 0.05, ∗∗p < 0.01, ∗∗∗p < 0.001, ∗∗∗∗p < 0.0001 (A and B Scale Bars: 250 μm), (C) qRT-PCR analysis of mRNA expression of IL1β, CD206 and IL10 (n = 4–8/group) Student's t-test. ∗∗p < 0.01 for comparison of male and female, #p < 0.05 for comparison with vehicle treatment. (D) CBA analysis of TNFα and IL 10 in liver tissue (n = 3–4/group), Student's t-test. ∗p < 0.05 for comparison of male and female mice, #p < 0.05 for comparison with vehicle treatment.

**Fig. S2:**
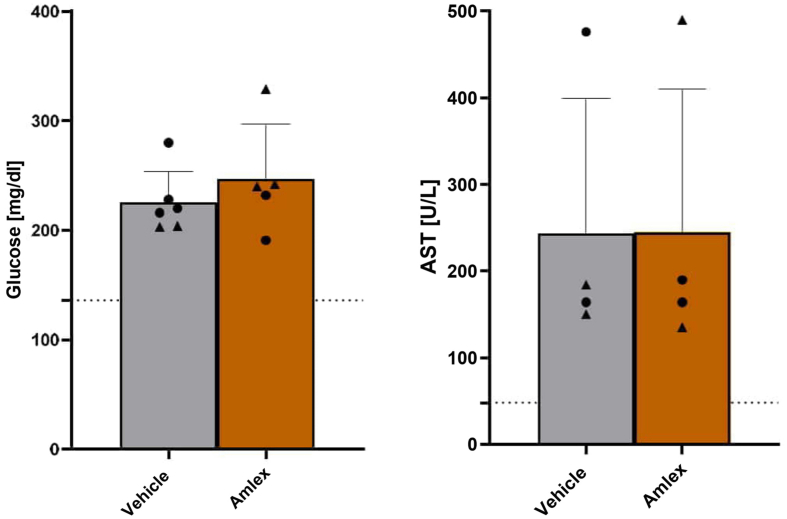
***Suppl. Figure 2:****Analysis of serum glucose and liver enzymes* Analysis of glucose and aspartate transaminase (AST) in the serum of male (●) and female (▲) mice after PCSK9/PD and vehicle or amlexanox treatment, respectively (n = 4–6). The dotted lines indicate the reference values.

The administration of PCSK9/PD to wild type mice resulted in a significant impact on the liver. Determination of serum glucose and the liver enzyme aspartate transaminase (AST) revealed elevated levels of all markers in comparison to reference values (glucose ∼136 mg/dL [31], AST ∼48 U/L [32]) which remained unchanged following treatment with amlexanox (glucose vehicle: 225 ± 26 mg/dL, amlexanox: 247 ± 45 mg/dL, AST vehicle: 244 ± 135 U/L, amlexanox: 339 ± 143 U/L) (Suppl. Figure 2). Nonetheless, a clear sex-dimorphism was evident in mice treated with amlexanox. Specifically, male mice exhibited a marked improvement in hepatic morphology, while female mice demonstrated a deterioration in liver appearance when compared to vehicle-treated mice. Liver morphology and ORO/H staining of liver slices exhibited severe fat deposition in female mice, a finding that was unaltered in mice administered amlexanox. In male control mice, liver morphology also showed fat depositions, but the content of ORO-stained lipid droplets was much lower than in female mice (p < 0.0001) and was significantly reduced by amlexanox administration compared to vehicle (Figure 3A). Immunohistology analysis of different immune cells in the liver revealed significant changes in Ly6G-, CD11b- and CD8b-positive cells between male and female mice. CD8b-positive T-cells were undetectable in both male control and amlexanox-treated mice; however, a significant increase was observed in female mice treated with amlexanox. Ly6G- and CD11b-positive cells were increased in male mice treated with amlexanox, while no changes were observed in female mice compared to the vehicle control. To further assess these regulations in the liver, RT-PCR and CBA analysis for pro- and anti-inflammatory markers were performed. The results showed significantly increased levels of IL1β in the livers of female mice and elevated anti-inflammatory markers such as CD206 and IL-10 in male mice on mRNA level. Further protein analysis by CBA supported these data by showing decreased TNFα and increased IL-10 levels in amlexanox-treated male mice indicating an upregulation of anti-inflammatory macrophages in male mice (Figure 3B,C and D).

**Figure 4:**
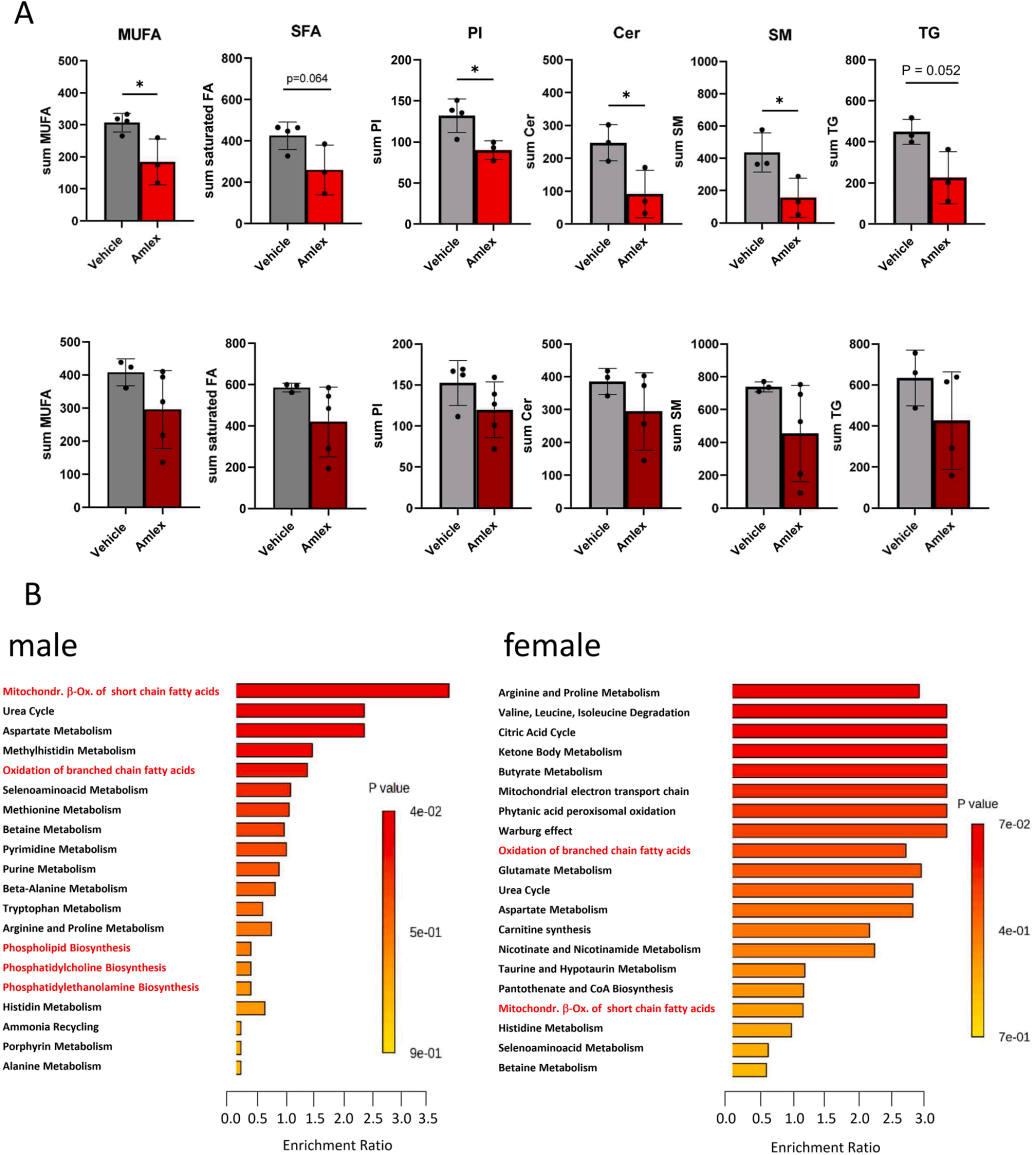
Lipid and metabolite analyses in serum of wild type mice treated with vehicle or amlexanox (A) Lipid regulations in serum were determined by Ultra High Performance Liquid Chromatography coupled to High Resolution Mass Spectrometry (UHPLC-HRMS). For analysis, peak ratios relative to an internal for all lipids of specific lipid classes were summarized for amlexanox- and vehicle-treated male (upper panel) and female (lower panel) wild type mice on PCSK9/PD, respectively (n = 3–5). Student's t-test. ∗p < 0.05 (MUFA: monounsaturated fatty acids, SFA: saturated fatty acids, PI: Phosphatidylinositol, Cer: Ceramides, SM: Sphingomyelins, TG: Triglycerides). (B) Enrichment analysis of metabolome data of serum of amlexanox- and vehicle-treated male and female wild type mice.

**Figure 5:**
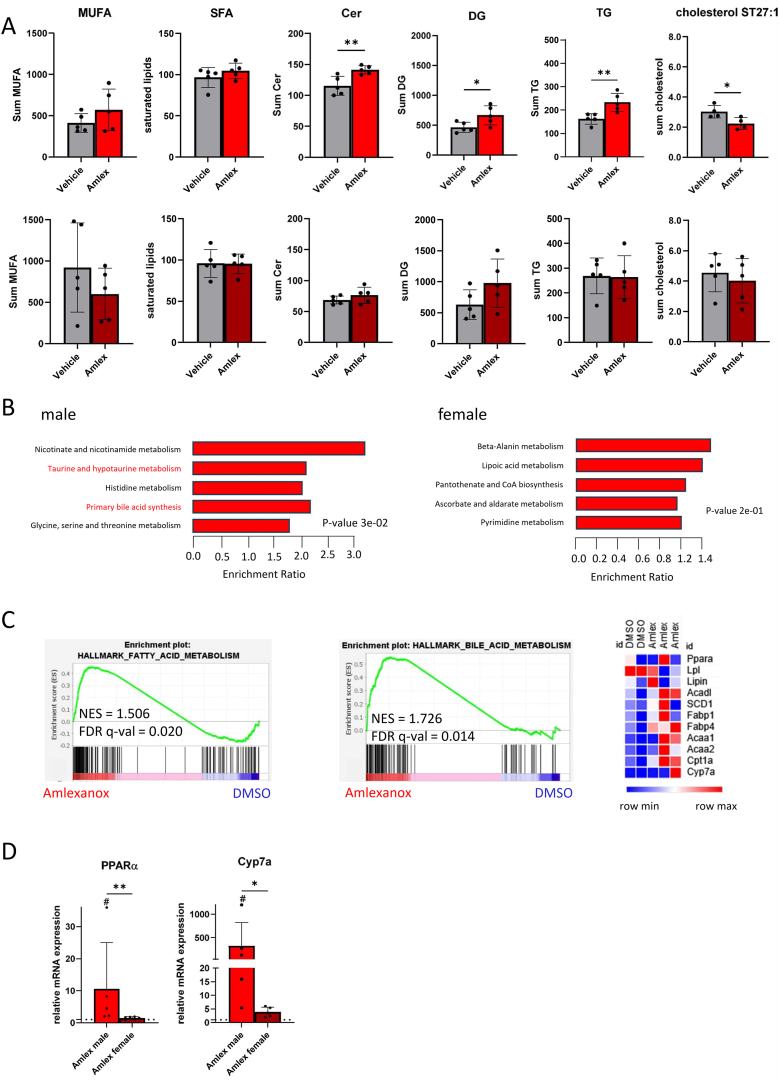
Lipid, metabolite and gene regulation in the liver of wild type mice treated with vehicle or amlexanox (A) Lipid regulations in the livers were determined by Ultra High Performance Liquid Chromatography coupled to High Resolution Mass Spectrometry (UHPLC-HRMS). For analysis, peak ratios relative to an internal for all lipids of specific lipid classes were summarized for amlexanox- and vehicle-treated male (upper panel) and female (lower panel) wild type mice on PCSK9/PD, respectively (n = 4–5). Student's t-test. ∗p < 0.05, ∗∗p < 0.01 (MUFA: monounsaturated fatty acids, SFA: saturated fatty acids, Cer: Ceramides, SM: Sphingomyelins, DG: Diacylglycerol, TG: Triglycerides). (B) Enrichment analysis of metabolome data of livers of amlexanox- and vehicle-treated male and female wild type mice. (C) Left side: Enrichment Plots from GSEA analysis of RNA sequencing data of livers of male wild type mice treated with PCSK9/PD and amlexanox or vehicle (n = 2–3/group). right side: heat map of representative genes involved in fatty acid and bile-acid metabolism, (D) mRNA regulations of PPARα and Cyp7a in the livers of male and female mice as assessed by RT-PCR analysis. Data were normalized against the respective vehicle treatment which was set as 1 for better comparison (dotted line), (n = 5–6/group). Student's t-test ∗p < 0.05, ∗∗p < 0.01.

**Fig. S3:**
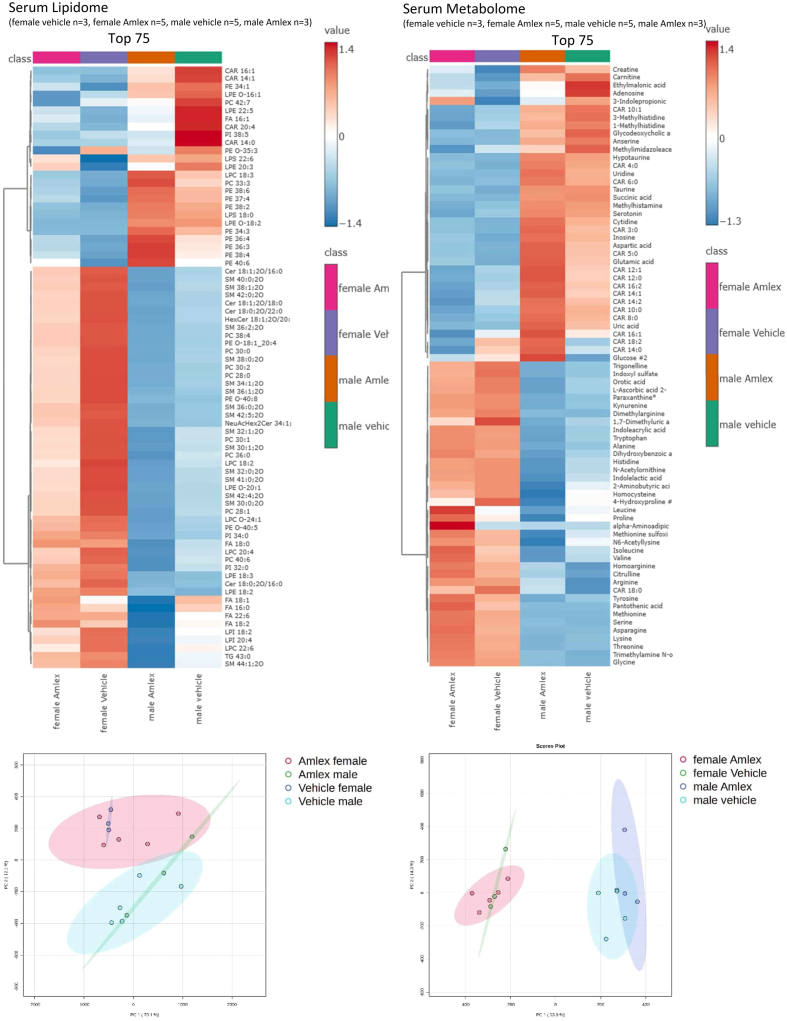
***Suppl. Figure 3:****Serum lipidome and metabolome analyses* Principal component analyses (PCA) and heatmaps (mean of 3-5 samples/group) of serum lipidome and metabolome analyses by high resolution mass spectrometry showing striking differences between male and female mice.

**Fig. S4:**
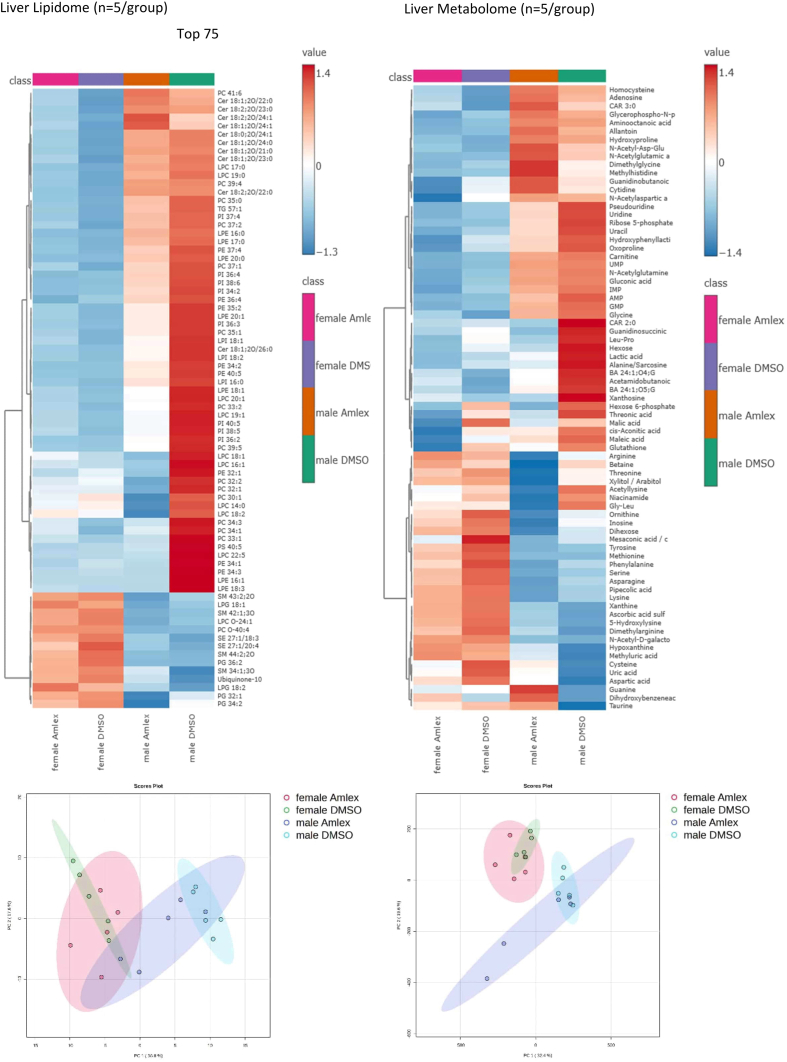
***Suppl. Figure 4:****Liver lipidome and metabolome analyses* Principal component analyses (PCA) and heatmaps (mean of 5 samples/group) of liver lipidome and metabolome analyses by high resolution mass spectrometry showing striking differences between male and female mice.

**Fig. S5:**
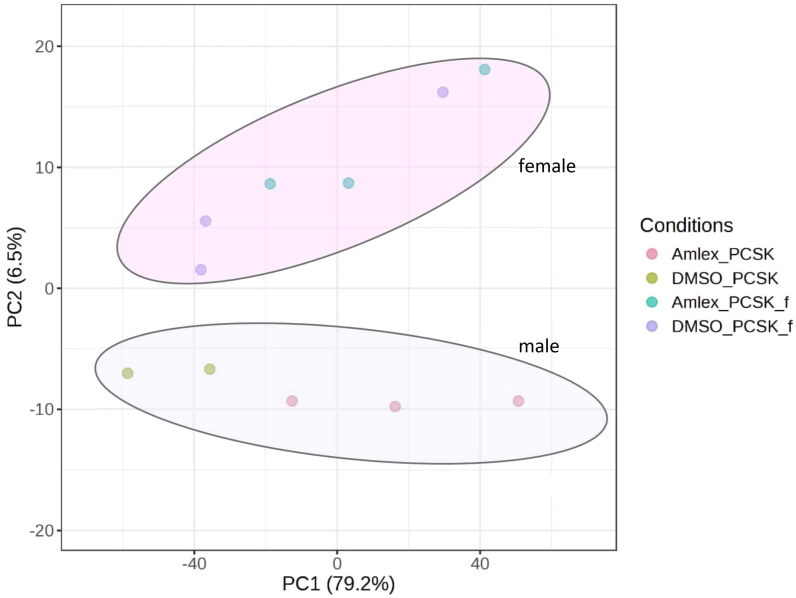
***Suppl. Figure 5:****Principal component analysis (PCA) of liver RNA sequencing*

Lipidomic and metabolomic evaluations were conducted to further assess the differential regulation of lipids and metabolites in the serum and liver tissue of male and female mice which might contribute to the observed differential appearance of the liver. In accordance with the apparent contrast in liver morphology, pronounced sex-specific differences were observed in the serum and liver lipidome and metabolome as indicated in PCA scores plots and heat maps (Suppl. Figures 3 and 4). A comprehensive analysis of diverse lipid categories revealed a significant downregulation of various types of lipids, including monounsaturated fatty acids (MUFA), saturated fatty acids (SFA), phosphatidylinositol (PI), ceramides (Cer), sphingomyeloids (SM), and triglycerides (TG) in the serum of male mice. The same trend was observed in female serum, albeit without statistical significance (Figure 4A). These results were supported by metabolome analysis, which indicated alterations in several fatty acid metabolic and phospholipid biosynthesis pathways in male mice that were much less pronounced in female mice (Figure 4B). Conversely, in the liver tissue of male mice, ceramides, diacyglycerols and triglycerides were found to be significantly elevated while free cholesterol was reduced. Liver tissue from female mice once more exhibited no regulatory differences in lipids between vehicle- and amlexanox-treated mice (Figure 5A). Enrichment analysis of the liver metabolome indicated a substantial regulatory impact on taurine metabolism and bile-acid synthesis in male mice (Figure 5B), a finding that aligns with RNA sequencing data. The sequencing results again demonstrated apparent differences between male and female mice (Suppl. Figure 5) as well as an enrichment of genes associated with fatty acid and bile-acid metabolism in male mice treated with amlexanox (Figure 5C). PCR analyses into the expression levels of genes involved in bile-acid synthesis in the liver tissue of male mice confirmed an upregulation of genes such as PPARα and Cyp7a (Figure 5D).

**Figure 6:**
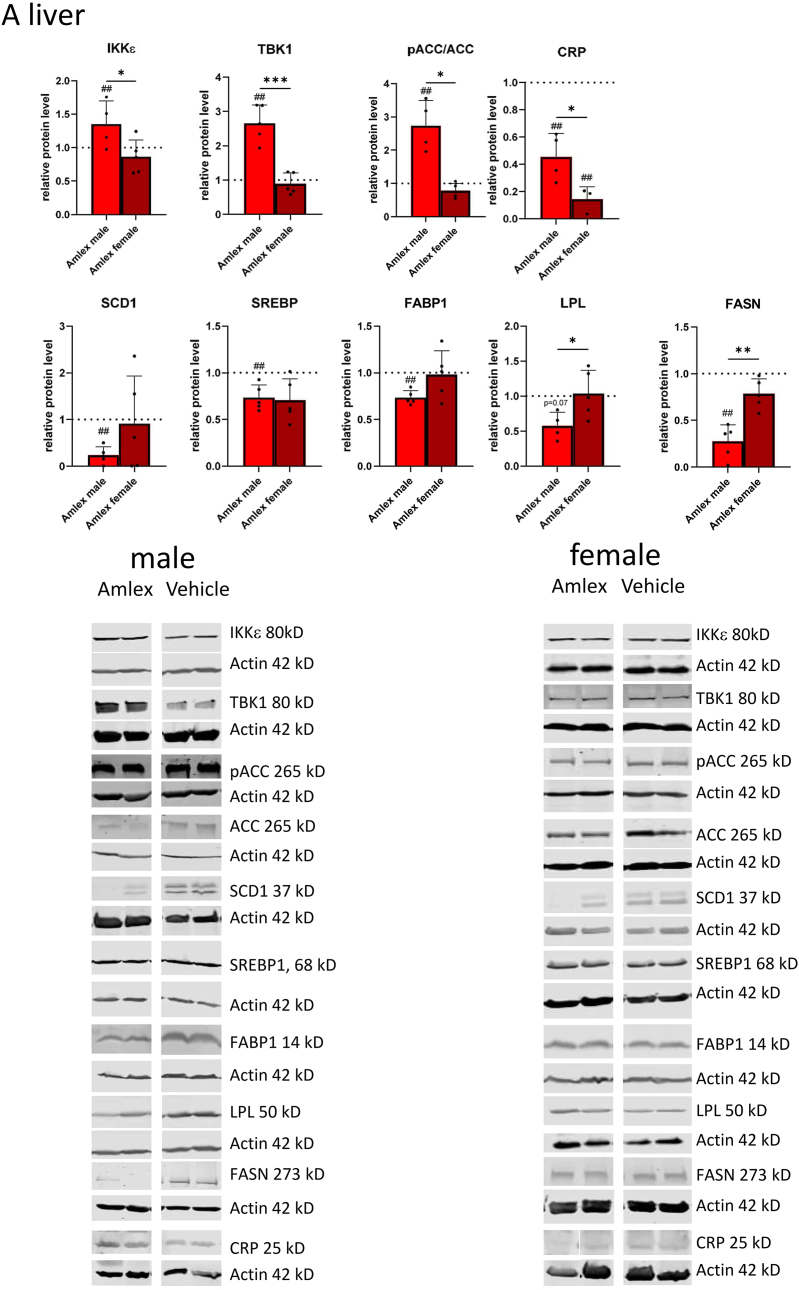

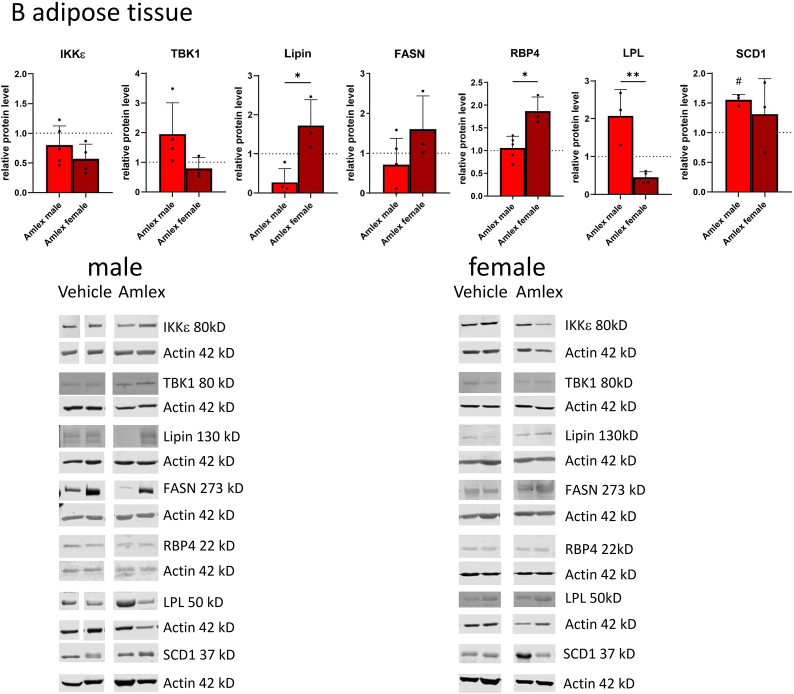
Protein regulations in the liver and adipose tissue of vehicle or amlexanox-treated mice (A) Western Blot analysis of different proteins in the livers of male and female wild type mice treated with PCSK9/PD and vehicle or amlexanox (n = 3–5). Protein bands were first normalized against the loading control β-actin and then against the respective vehicle control mice. (B) Western Blot analysis of different proteins in the WAT (n = 3–6). Protein bands were first normalized against the loading control β-actin and then against the respected vehicle control mice. The dotted lines in the diagrams indicate the respective vehicle groups, which were set as 1 for better comparison. Representative Blots for each protein are shown below the quantitative analysis. Student's t-test. ∗p < 0.05, ∗∗p < 0.01, ∗∗∗p < 0.001, p < 0.0001, significant differences in comparison of male and female mice, #p < 0.05, ##p < 0.01 significant differences in comparison to the vehicle control.

**Fig. S6:**
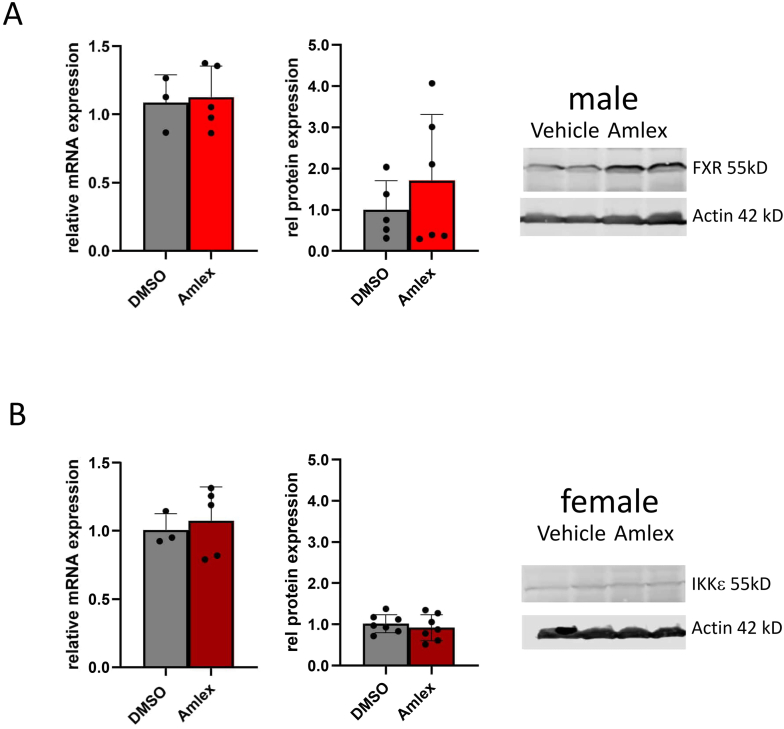
***Suppl. Figure 6:****Regulation of liver FXR* mRNA and protein expression of FXR in the liver of (A) male and (B) female mice treated with either vehicle or amlexanox. The left panels show the qRT‑PCR results (n = 3–5 per group), and the middle panels present the Western blot quantification (n = 5–7 per group). For comparability, vehicle controls were set to 1. The right panels display representative Western blot images.

To further investigate the observed differences at the protein level, Western Blot experiments were performed on liver and adipose tissue samples. While female mice demonstrated less modulation in comparison to the vehicle control mice, the livers of male mice exhibited several differential regulations compared to vehicle and also to female mice. ΙΚΚε and TBK1 were both found to be increased in male mice, with significantly higher expression levels than those observed in female mice. Furthermore, phosphorylation of ACC, the substrate for AMP-activated kinase, was significantly enhanced in male mice with no change in female mice. C-reactive protein was significantly reduced in both amlexanox-treated male and female mice with a stronger reduction in females. These changes might be attributed to an anti-inflammatory response after amlexanox treatment. Protein levels of SCD1, SREBP, FABP1, FASN and LPL were significantly decreased in male mice in comparison to the vehicle control group, respectively (Figure 6A). These regulations may contribute to a reduction in lipid levels by amlexanox (Figure 3, Figure 4), thereby leading to an improvement in MASLD. FXR levels in the liver were also examined and showed no differences between livers from amlexanox- and vehicle control-treated animals (Suppl. Figure 6).

In adipose tissue, no significant regulations of ΙΚΚε and TBK1 were observed in either sex. Lipin, FASN, and RBP4 levels were elevated in female mice, while LPL levels were found to be significantly increased in male mice and decreased in female mice (Figure 6B).

## 4 Discussion

In a recent study, we could demonstrate that genetic deletion of IKKε markedly reduced atherosclerosis initiation and progression and improved MASLD in male, but not female mice [19]. Here, we evaluated the therapeutic efficacy of pharmacological IKKε inhibition using amlexanox in mice with established atherosclerosis and hepatic steatosis. Our data showed that, although the late-stage use of amlexanox attenuated further increases in circulating lipids, it neither halted nor reversed atherosclerosis progression and did not reduce plaque size in either sex. However, plaques of male mice displayed lower lipid deposition and a reduced number of CD86-positive cells like e.g. macrophages. Sex-specific effects were also evident in the liver with male amlexanox-treated mice showing improved liver morphology, reduced serum lipid concentrations, and altered bile-acid synthesis, whereas females did not benefit. It is important to note that the diet alone already induced pronounced sex-specific differences, with female mice developing more severe MASLD than male mice. This supports the interpretation that amlexanox may effectively counteract disease progression at lower levels of hepatic fat accumulation, but is not sufficient to reverse more advanced stages of the disease.

Previous studies using a high fat diet model in wild type mice reported reduced weight gain and liver steatosis and improved insulin sensitivity by an upregulation of energy expenditure after amlexanox treatment. These effects were associated with an inhibition of liver and adipose tissue inflammation [21] as well as molecular regulations in hepatic stellate cells [33]. Zhao et al. treated male LDL-receptor knock-out mice (LDL-R−/−) with a HFD and showed that amlexanox mitigated atherosclerosis and reduced cholesterol levels by increased bile-acid excretion mediated by an upregulation of Cyp7 levels [18]. In a follow-up study, therapeutic administration of amlexanox in long-term HFD-fed LDL-R^−/−^ mice also reduced liver injury, fibrosis, and inflammation as well as attenuated atherosclerosis progression [23]. Our study combined PCSK9 overexpression and a Paigen diet in wild type mice which differs substantially from standard HFDs in fat content, duration, and the inclusion of sodium cholate. The Paigen diet is considered as non-obese diet due to relatively low fat content but the addition of sodium cholate enhances lipid absorption, and exacerbates hepatic steatosis and cholestasis [34]. Consequently, the Paigen diet induces rapid and robust atherosclerotic plaque formation [[35], [36], [37]]. In addition, sodium cholate is an activator of the nuclear receptor FXR [38], one of the main regulators of bile-acid synthesis. FXR activation suppresses cholesterol 7α-hydroxylase Cyp7a, the rate-limiting enzyme of the classical bile-acid synthesis pathway, thereby reducing bile-acid production while increasing hepatic cholesterol levels [29,30]. These effects were also evident in our control mice on control and Paigen diet. The interplay between FXR and estrogen receptor alpha (ERα) has been proposed to result in mutual inhibition of ERα- and FXR-dependent signaling, potentially contributing to the sex-specific differences observed in MASLD [39]. Since Cyp7a expression was increased only in male mice treated with amlexanox, this might indicate a sex-specific interaction between FXR and IKKε.

The model-specific features likely explain why amlexanox improved serum lipid profiles and plaque composition in our study but did not reduce weight gain, glucose levels and plaque size, in contrast to findings from HFD-based models. Furthermore, it might support the pronounced sex-specific effects. In general, the composition and duration of diet and treatment was different among the published reports (Table 1), making direct comparison difficult.

**Table 1:** Comparison of the treatment strategies of mice in different studies.

Mice	*C5*7BL*/6J wild type treated with PCSK9 GOF*	*C5*7BL*/6J wild type*	*LDL-R^−/−^*	*LDL-R^−/−^*	*C5*7BL*/6J wild type*
Diet	Paigen diet (Ssniff EF D12336 mod.) 4.1% Sugar 16% fat 1.25% cholesterol, 0.5% sodium cholate	High fat diet research diets D12492 60% fat 10% sucrose	High fat diet (research diets D12079Bi) 21% sucrose 35% fat 0.15% cholesterol	GAN diet (research diets D09100310) 40% fat 20% Fructose 10% sucrose 2% cholesterol	HFD research diets 12451 45% fat 20% sucrose
Start of treatment after diet start	8 weeks	Directly	3 weeks or 8 weeks	20 weeks	Directly or 12 weeks
Duration drug treatment	8 weeks	18 weeks	8 weeks or 4 weeks	12 weeks	12–24 weeks
Ref.	This manuscript	[33]	[18]	[23]	[21]

In the liver, amlexanox improved liver steatosis and reduced inflammatory markers in male mice while female mice exhibited increased inflammation. Although the upregulation of CD11b- and Ly6G-positive cells in liver tissue in male mice may indicate an increased inflammatory response, transcriptomic data suggested a shift toward anti-inflammatory phenotypes that promote the resolution of inflammation. Serum lipidomics revealed broad reductions in saturated and unsaturated fatty acids, triglycerides, sphingomyelins, and ceramides in males, consistent with decreased de novo lipogenesis and protection against steatosis [40]. Interestingly, hepatic lipid species increased in male mice despite improved histology. Although these lipid regulations are often discussed for their debilitating properties in MASLD [41,42], this pattern may also reflect enhanced lipid utilization, microvesicular storage, or reduced VLDL secretion, mechanisms that can prevent harmful macrovesicular droplet formation and thereby protect against liver injury [[43], [44], [45]]. Reduced hepatic free cholesterol further supports improved liver health [46]. Female mice showed no significant changes of serum and hepatic lipids after amlexanox-treatment.

Metabolome and transcriptome analyses indicated an enrichment of genes and metabolites involved in fatty acid oxidation and bile-acid metabolism in the liver of male mice, including an upregulation of PPARα and Cyp7a which is consistent with earlier reports [18,23]. Analyses of protein-levels in the liver corroborated these findings. Increased levels of phosphorylated Acetyl-CoA-carboxylase (pACC) hint to reduced fatty acid synthesis and inflammation [47,48], accompanied by downregulation of FASN, SCD1, and SREBP, key regulators of lipogenesis [[49], [50], [51]]. Reduced SCD1 expression is associated with enhanced fatty acid oxidation and lower circulating lipids, aligning with our observations [52]. Upregulation of ΙΚΚε and TBK1 likely reflects compensatory feedback regulation following pharmacological inhibition [21,22].

## 5 Conclusion

In summary, our data indicate that therapeutic IKKε inhibition with amlexanox does not reverse advanced atherosclerosis but can partially improve plaque composition. In contrast, established MASLD is ameliorated in male mice through modulation of lipid and bile-acid metabolism. The absence of beneficial effects in females highlights a critical sex-specific divergence that has been largely overlooked in previous studies, which predominantly used male animals. These findings underscore the importance of incorporating both sexes in preclinical and clinical evaluations of amlexanox as a potential therapy for MASLD.

## CRediT authorship contribution statement

**Eleonora Mungo:** Writing – review & editing, Methodology, Investigation, Formal analysis, Data curation. **Michelle Haß:** Writing – review & editing, Methodology, Investigation. **Denis Benning:** Methodology, Investigation. **Tobias Schmid:** Writing – review & editing, Supervision, Methodology. **Silvia Kuntschar:** Writing – review & editing, Methodology. **Sofie P. Meyer:** Writing – review & editing, Methodology, Conceptualization. **Lisa Hahnefeld:** Writing – review & editing, Supervision, Methodology, Formal analysis, Data curation. **Erika Dorochow:** Writing – review & editing, Methodology, Formal analysis, Data curation. **Urs Christen:** Writing – review & editing, Methodology. **Edith Hintermann:** Writing – review & editing, Methodology. **Rebekka Medert:** Writing – review & editing, Resources. **Marc Freichel:** Writing – review & editing, Resources. **Gerd Geisslinger:** Writing – review & editing, Resources. **Ellen Niederberger:** Writing – original draft, Supervision, Project administration, Methodology, Investigation, Funding acquisition, Formal analysis, Data curation, Conceptualization.

## Ethics declaration

This study was conducted in accordance with the ARRIVE (Animal Research: Reporting of In Vivo Experiments) guidelines. This study was approved by the Regierungspräsidium Darmstadt. (Approval No. FK/1004 and FK/1115)

## Declaration of competing interest

None of the authors has a known competing financial interest/personal relationships which may be considered as potential competing interests: Ellen Niederberger reports financial support was provided by German Research Foundation. If there are other authors, they declare that they have no known competing financial interests or personal relationships that could have appeared to influence the work reported in this paper.

## Data Availability

Data will be made available on request.
