# Supplementary material for: Sex-specific differences of amlexanox in a mouse model for atherosclerosis and MASLD
Source: Mol Metab. 2026 Jul 21;111:102426. doi: 10.1016/j.molmet.2026.102426 (PMC13453042; doi:10.1016/j.molmet.2026.102426)
Supplement: Supplementary file 1 [file mmc1.docx]

# **Supplemental Figures**

***Suppl. Figure 1:*** *Analysis of serum and liver parameters*

(A) Correlation of serum amlexanox concentration with serum LDL-cholesterol levels in male and female mice (n=3 for male, n=4 for female mice). (B) Total Cholesterol, LDL- and HDL-cholesterol and triglycerides in the serum of female and male wildtype mice after PCSK9 injection and eight weeks of Paigen diet. Control animals received a NaCl injection and Paigen diet for eight weeks. The injection of PCSK9 induced significantly higher lipid levels compared to NaCl-treated mice. (n= 4-9/group), One-way ANOVA, ** p < 0.01,*** p < 0.001,**** p < 0.0001. (C) FXR protein level assessed by Western Blot analysis, Cyp7a mRNA expression in the liver and serum total bile-acid levels of male and female control mice treated with either control (CD) or Paigen diet (PD). Student’s t-test, *p < 0.05 (n = 2-3/group)

***Suppl. Figure 2:*** *Analysis of serum glucose and liver enzymes*

Analysis of glucose and aspartate transaminase (AST) in the serum of male (●) and female (▲) mice after PCSK9/PD and vehicle or amlexanox treatment, respectively (n = 4–6). The dotted lines indicate the reference values.

***Suppl. Figure 3:*** *Serum lipidome and metabolome analyses*

Principal component analyses (PCA) and heatmaps (mean of 3-5 samples/group) of serum lipidome and metabolome analyses by high resolution mass spectrometry showing striking differences between male and female mice.

***Suppl. Figure 4:*** *Liver lipidome and metabolome analyses*

Principal component analyses (PCA) and heatmaps (mean of 5 samples/group) of liver lipidome and metabolome analyses by high resolution mass spectrometry showing striking differences between male and female mice.

***Suppl. Figure 5:*** *Principal component analysis (PCA) of liver RNA sequencing*

***Suppl. Figure 6:*** *Regulation of liver FXR*

mRNA and protein expression of FXR in the liver of (A) male and (B) female mice treated with either vehicle or amlexanox. The left panels show the qRT‑PCR results (n = 3–5 per group), and the middle panels present the Western blot quantification (n = 5–7 per group). For comparability, vehicle controls were set to 1. The right panels display representative Western blot images.
